# Designing and implementation of a mobile application for teaching population oral health needs assessment for dental students; a non-randomized trial

**DOI:** 10.1038/s41405-024-00287-4

**Published:** 2024-12-22

**Authors:** Hadi Ghasemi, Amin Habibi, Soleiman Ahmady

**Affiliations:** 1 Department of Community Oral Health, School of Dentistry, Shahid Beheshti University of Medical Sciences, Tehran, Iran; 2 Department of Medical Education, Virtual School of Medical Education and Management, Shahid Beheshti University of Medical Sciences, Tehran, Iran

**Keywords:** Dental clinical teaching, Dental epidemiology

## Abstract

**Objective:**

This study investigates the effectiveness of a newly developed smartphone-based application for teaching population oral health needs assessment to undergraduate dental students.

**Methods:**

Target population in this study consisted of all students of Shahid Beheshti School of dentistry in the 7^th^ and 8^th^ semesters in the year 2023. The intervention group (7^th^ semester) received teaching about population oral health needs assessment based on the book “Oral health surveys; basic methods”, by means of an application, while the control group (8^th^ semester) received the same content through self-learning activity. A questionnaire inquiring about the students’ general aspects of smartphone usage, attitude towards learning based on mobile devices (m-learning), and level of knowledge about the content of the book was used for data collection.

**Results:**

Most dental students used smartphones extensively and expressed positive attitudes toward mobile learning with no significant statistical difference between intervention and control groups. However, both groups demonstrated limited knowledge gain from the book content, with the mobile application showing no superiority to self-learning education.

**Conclusion:**

The present study did not demonstrate superior effectiveness of a smartphone app compared to traditional teacher-centered instruction, but the students’ significant mobile usage and positive attitude towards m-learning suggest potential for further investigation in dental education.

## Introduction

In response to the growing social needs for education and limited resources for traditional education, the use of communication and information technology tools, including mobile phones, has grown significantly in recent years. Mobile applications, as technologies that are present in all aspects of life, have been shown to facilitate education [1]. Healthcare students usually use them for tasks such as documentation, time management, health record management, consulting, and networking with their teachers, finding scientific information, acquisition and reading references, patient’s care and monitoring, and clinical decision making.

The use of mobile applications in dental education is very wide and increasing at a significant rate [2]. Dental students generally use mobile applications for the educational purposes [3, 4]. It is presumed to be a huge potential for the development of academically beneficial apps relating to dentistry [5]. Mobile applications were found to be effective in the improvement of dental students’ knowledge and competency in different aspects of dental education such as practical pathology [6], management of dental trauma injuries [7], use of laser in dentistry [8], radiographic diagnosis of endodontic problems [9], differential diagnosis of maxillofacial bony lesions [10], and recording and evaluating the oral health status for a population [11]. Continuing professional development programs has also benefited from learning applications based on smartphones [12] especially after limitations of conventional education due to COVID-19 pandemic [13].

The first step for any health promotion activity at the community level is health need assessment. For this purpose, one of the basic subjects that is included in the curriculum of undergraduate dental curriculum is the topic of “population oral health need assessment” which requires dental students to efficiently register various oral health indicators among individuals and populations. This topic is taught as a subject in the community oral health course in Iran. Among the sources used to teach this topic is the “Oral health surveys; basic methods” [14], a publication of World Health Organization, in which a simple yet accurate method to assess the oral health status of the population is recommended. In addition to being an educational resource for dental students, the book is used and cited as a standard and internationally recognized method in most research related to the oral health of populations. A look at the articles related to the oral health assessment of the population in the relevant scientific literature shows the wide use of the recommended method in this book. For example, in the most recent national oral health surveys in Iran in 2012 [15] and 2017 [16], the method suggested in the book were employed. It is expected that employing an educational application that includes different conditions of oral and dental tissues in the form of photos, images, and text, could facilitate the process of teaching-learning for this method. Furthermore, the integration of mobile-based educational applications can also address some of the challenges faced by dental students in traditional lecture-based education. One such challenge is the limited opportunity for hands-on practice and real-time feedback [17]. Mobile applications can provide virtual simulations and case-based scenarios, allowing students to practice different dental procedures and receive immediate feedback on their performance.

The Kirkpatrick model is an evaluation model used to assess the effectiveness of educational programs [18]. It consists of four levels: reaction, learning, behavior, and impact. At the reaction level, the model aims to determine the participants’ response to the program. The learning level assesses the extent of knowledge, attitude, skills, and behavior change in the participants. The behavior level evaluates the changes in participants’ behavior because of the program. Finally, the impact level examines the overall outcomes and results of the program. Several studies have utilized the Kirkpatrick model to evaluate the effectiveness of educational apps. For example, Carnell et al. employed Kirkpatrick model as a framework to evaluate educational and training applications using virtual environments [19]. Another study by Banasr et al. evaluated a dental sleep medicine mini-residency continuing education program using the Kirkpatrick model [20] and showed a positive impact on training, with high levels of participant satisfaction, increased knowledge scores, and successful transfer of knowledge and skills to practice. These studies demonstrate the applicability of the Kirkpatrick model in evaluating the impact of mobile applications. By considering the levels of satisfaction, learning, behavior, and results, the Kirkpatrick model provides valuable insights for improving the quality and effectiveness of dental education apps.

This study aimed, therefore, to evaluate the effectiveness of a newly developed smartphone-based application for teaching population oral health needs assessment to the undergraduate dental students of Shahid Beheshti University of medical sciences. It is hypothesized that the application can enhance the students’ level of knowledge, attitude, and practice regarding oral health needs assessment based on Kirkpatrick educational model.

## Methods

The current research adheres to the principles outlined in the CONSORT guideline [21]. In this non-randomized experimental study, the target population included all students of Shahid Beheshti School of dentistry in the 7^th^ semester as the intervention and in the 8th semester as the control group in the year 2023. The intervention group received the topic of oral health needs assessment by means of a mobile application and the control group had received the same topic as a self-learning activity. The educational content was the same in both methods and in accordance with the undergraduate dental curriculum.

Based on the undergraduate dental curriculum in Iran, the topic of “population oral health needs assessment” is taught in the community oral health course. The reference for teaching this topic is a manual entitled “Oral health surveys; basic methods” [14], a publication of the World Health Organization, in which the detailed explanation of standard oral health evaluation forms, different variables, measurement criteria and their options are provided. Essentially in this topic, dental students are expected to get familiar with the following two types of assessment forms during this course: a clinical examination sheet and a questionnaire inquiring about individuals’ oral health behaviors, the effect of oral health status on quality of life and some socio-economic variables affecting oral health.

In the clinical examination sheet, standardized forms for recording clinical oral health assessments are provided separately for adults and children. Each form includes more than 200 variables, and the questionnaire covers about 50 variables which are expected to be filled by dental students. Standard codes must be used for all sections of the forms otherwise processing the data and summarizing the results will be problematic. In addition, the manual includes 72 illustrations regarding to the major oral conditions which may be helpful in differential diagnosis of lesions, increasing accuracy of coding during the examination.

In the control group, students were provided with the book in a PDF format and were instructed to collaborate on translating specific sections of the book as part of group assignments. They were then tasked with creating a PowerPoint presentation based on their translations, presenting it to the entire class, and engaging in discussions under the guidance of the instructor. Subsequently, during the following session, students were given printed clinical forms and questionnaires. They were required to fill out these forms for two of their peers during an authentic dental examination using a single-use dental mirror in natural light. The instructor collected the completed forms for further evaluation and feedback provision.

In the intervention group, on the other hand, students received a link to download a specifically designed application on their smartphones. The application has been designed as a Rest API for a web version using Asp.net Core technology. For the back-end, C#.net, and for the front-end, Angular js, jQuery, HTML, and CSS have been employed. In the design and implementation of the database, Microsoft SQL Server 2019 has been employed incorporating stored procedures. The mobile version that was compatible with both Android and iOS platforms was implemented using Flutter. The mobile version interacted with the server by connecting to Web APIs for data exchange. The application contained the PDF version of the manual, allowing students to read it and obtain Persian translations when needed. Table 1 shows more details on different pages of the application. Like the control group, students in the intervention group were tasked with conducting dental examinations and completing questionnaires for two classmates. However, they entered the data directly into electronic forms and questionnaires within the application. The results were then submitted to the instructor for evaluation and feedback. The current study faced some administrative challenges that made it impossible to perform blinding for the students, the instructor, or the statistician. To compare these two educational methods, a questionnaire was employed to evaluate the level of the students’ knowledge at three stages as follows: for the intervention group, before the start of teaching (February 2023; pre-test) and after the end of teaching (July 2023; post-test), and for the control group just after the end of teaching (February 2023; post-test). The students’ average scores out of the different parts of the questionnaire were compared among the experimental group (pre- and post-test) and between experimental and control groups (post-test).

**Table 1:** Content presented in different pages of the application.

Screen No.	Content
Screen 1	Home screen with main menu options.
Screen 2	The first page introduces topics related to population oral health needs assessment, based on the WHO manual. These topics include clinical oral health assessments and behavioral questionnaires.
Screen 3	Selecting the “Clinical Examination Sheet” option opens a page displaying separate forms for adults and children.
Screen 4	The “Oral Health Behavior Questionnaire” option provides a page asking about oral health behaviors, the impact of oral health on quality of life, and socio-economic factors.
Screen 5	The form completion screen allows students to enter data using standardized codes for each section of the clinical examination form and questionnaire. Incorrect codes trigger prompts for correction.
Screen 6	The “Illustrations for Diagnosis” screen contains 72 images showing common oral health conditions, assisting students in the differential diagnosis and accurate coding.
Screen 7	The “Translation Support” screen provides access to the PDF of the WHO manual with Persian translation features, allowing students to toggle between languages as needed.
Screen 8	The “Data Submission” page allows students to submit completed clinical forms and questionnaires electronically for evaluation and feedback from the instructor.
Screen 9	The “Instructor Feedback” screen displays feedback from the instructor after evaluation, highlighting errors and providing suggestions for improvement.
Screen 10	The “Knowledge Assessment” screen presents pre- and post-tests to evaluate students’ understanding of the material, with immediate scoring and feedback.

The questionnaire included the following sections: fifteen questions inquiring about the students’ background characteristics and general aspects of smartphone usage (Tables 2, 3), adopted with minor modifications from Koopaie et al. [22], twenty one questions about the students’ attitude towards learning based on mobile devices (m-learning) [23] (Table 4), and fifteen multiple choice questions (MCQ) to assess the students’ level of knowledge about the content of the book (Table 5). The latter MCQs were formulated by one of the authors (HG) with 15 years of experience in teaching the topic of “oral health needs assessment for the population” to general dental students. Face and content validity of the questionnaire was, moreover, discussed with and ultimately approved by all authors who are professors in the field of dental public health and medical education. The reliability coefficient (Cronbach alpha) for the relationship between items of the first two sections of the questionnaire was close to 0.6. The Chi-square test, Mann-Whitney U test, Independent sample T-test were employed for the statistical analysis.

The answers to different parts of the questionnaire were organized as follows: in the section focused on general mobile phone usage, students were asked to choose the option that most accurately represented their level of agreement with each question from a five-point Likert scale (very much, much, medium, few, and very few). For the attitude section, the participants were requested to express their degree of agreement with each statement regarding attitudes towards learning through mobile phones, utilizing a five-point Likert scale ranging from complete agreement (score of 5) to complete disagreement (score of 1). The cumulative score derived from 21 statements, with a possible theoretical range of 21 to 105, was interpreted as the respondents’ overall attitude, where higher scores reflected a more positive attitude. For the knowledge assessment questions, students received points for each question they answered correctly from a set of four possible answers.

The study was approved by the Ethics Committee of the Shahid Beheshti School of Dentistry under the ethical code IR.SBMU.DRC.REC.1400.081. The students’ agreement to complete the questionnaire was considered as informed consent for participation in the research. They were, also, guaranteed that their answers would be kept confidential and utilized solely for research purposes. This assurance was included in the introductory explanation of the questionnaire and reiterated when the questionnaire was distributed to them.

## Results

A total of 104 completed questionnaires were collected from the students; however, six were excluded due to incomplete responses. This resulted in a final dataset of 98, which served as the basic data for this study; more than half of them were men. As depicted in Table 2, over 60% of the students reported utilizing their smartphones for more than three hours daily.

**Table 2:** Distribution (%) of the students (*n* = 104), based on their background factors.

		All (%)	Study groups		
			Control (%)	Intervention (%)	*P*-value*
Gender	Male	59 (57)	34 (56)	25 (58)	0.84
	Female	45 (43)	27 (44)	18 (42)	
Age (years)	≤ 23	83 (80)	47 (77)	36 (84)	0.46
	>23	21 (20)	14 (23)	7 (16)	
Use of mobile (hours/day)	≤ 3	41 (39)	22 (35)	19 (44)	0.42
	> 3	64 (61)	40 (65)	24 (56)	

*Statistical evaluation by the Chi-square test.

Table 3 presents students’ answers to general questions on using mobile applications. Majority of the respondents agreed with producing a dental educational mobile app and liked to use such an app. Moreover, most of the respondents were satisfied with the subject of oral health registration in the course and evaluated their learning good, felt it necessary to produce a mobile app for the oral health registration, and desired to use such a mobile app, with no statistically difference between control and intervention groups.

**Table 3:** Distribution (%) of the students’ (n = 98) answers regarding to use of mobile applications.

		All (%)	Study groups		
			Control (%)	Intervention (%)	*P*-value^a^
How frequent do you use mobile apps?	≥ medium^b^	79 (81)	46 (74)	33 (92)	0.04
	< medium^c^	19 (19)	16 (26)	3 (8)	
How much do you agree with producing a dental educational mobile app?	≥ medium	91 (93)	57 (92)	34 (94)	1.00
	< medium	7 (7)	5 (8)	2 (6)	
How much do you like to use a dental educational mobile app for dental courses?	≥ medium	92 (94)	58 (94)	34 (94)	1.00
	< medium	6 (6)	4 (6)	2 (6)	
How much do you satisfy with oral health registration according to WHO in the course COH2?	≥ medium	81 (84)	48 (79)	33 (92)	0.15
	< medium	16 (16)	13 (21)	3 (8)	
How do you evaluate your learning about oral health registration according to WHO in the course COH2?	≥ medium	78 (80)	47 (76)	31 (86)	0.30
	< medium	20 (20)	15 (24)	5 (14)	
How much do you feel necessary to produce a mobile app for the oral health registration according to WHO?	≥ medium	86 (88)	55 (89)	31 (86)	0.75
	< medium	12 (12)	7 (11)	5 (14)	
How much do you desire to use such a mobile app?	≥ medium	86 (88)	55 (89)	31 (86)	0.75
	< medium	12 (12)	7 (11)	5 (14)	

^a^Statistical evaluation by the Chi-square test.

^b^Selecting “very much”, “much”, and “medium” from the five-point Likert scale.

^c^Selecting “few” and “very few” from the five-point Likert scale.

The students’ attitude regarding m-learning are reflected in Table 4. Nearly three out of four of the students, with no significant statistical difference between intervention and control groups, believed that m-learning is beneficial, useful outside classrooms, useful to fill spare time, facilitate fast learning, improvement of research skills, self- learning, rapid feedback, easier storage of information, provide freedom of learning, and act as a compensation for missed classes.

**Table 4:** Percentages of the students’ (n = 98) agreement (selecting options agree and completely agree from the five-point Likert scale) with the statements regarding attitude towards mobile-based learning.

Item	Total	Control	Intervention	*p*-value*
I think m-learning is the most suitable environment for students with different learning styles (visual, auditory, learning by doing and experiencing, etc.).	65	57	77	0.07
I think that m-Learning can be more beneficial when it is combined with face to face learning in university courses. …	88	87	89	1.00
I think that m-Learning provides fast and practical learning.	81	82	80	0.79
I agree that m-Learning provides permanent learning.	60	57	66	0.51
I believe that it would be useful to spare my free time (Bus waiting, rest, etc.) with m-Learning outside of the course.	78	74	86	0.21
I find it interesting m-learning since I don’t want to carry books and course materials.	83	83	83	1.00
I think m-Learning is a good opportunity to improve my research skills.	76	73	79	0.62
Due to the potential dangers of the Internet (virus, etc.), I think m-Learning environment is unsafe.	9	7	14	0.28
I think mobile devices are not suitable for use in m-Learning environment since they need to be charged regularly.	12	10	14	0.52
I think m-Learning applications outside the classroom are useless because my attention is easily dispersed on the move.	21	22	20	1.00
I do not find it appropriate to use mobile devices in the classroom because they are harmful to human health.	14	15	12	0.76
I think m-Learning is not suitable for courses that require more reading and writing.	34	33	35	0.82
I like to participate in m-Learning as I can access course materials faster.	76	76	75	1.00
I believe that m-Learning supports planned and systematic study.	64	62	67	0.66
I believe that mobile tools are useful for taking notes in class.	56	47	69	0.05
I think mobile tools are useful for storing information.	90	87	94	0.31
I believe that mobile devices with large screens are useful for m-learning.	84	80	92	0.16
I think that m-Learning is an appropriate method for courses that require individual effort.	75	74	78	0.81
I prefer to learn m-Learning because I can compensate myself for the lessons I have missed.	78	74	83	0.33
I believe that m-Learning is useful for getting rapid feedback.	75	71	81	0.34
I believe that using m-Learning in the courses at the university will increase my freedom of learning.	74	71	78	0.63

*Statistical evaluation by the Chi-square test.

Table 5 presents the answers of the students regarding 15 multiple choice questions from the content of the book. In total, 7-60% of the students answered correctly on questions and mean score of correct answers were 4.81 ( ± 2.16) out of theoretically 0-15, with no statistically significant difference between intervention and control groups.

**Table 5:** Frequency of the students’ (n = 98) correct answer and mean score from a list of fifteen multiple choice questions of the book “WHO oral health surveys basic methods; 5^th^ edition, 2013”.

Item	Total n (%)	Control n (%)	Intervention n (%)	*p*-value*
1	7 (7)	6 (10)	1 (3)	0.25
2	59 (60)	37 (60)	22 (61)	1.00
3	10 (10)	8 (13)	2 (6)	0.32
4	24 (25)	10 (23)	14 (28)	0.63
5	32 (33)	21 (34)	11 (31)	0.82
6	22 (22)	12 (19)	10 (28)	0.45
7	33 (34)	21 (34)	11 (33)	1.00
8	54 (55)	35 (57)	19 (53)	0.83
9	48 (49)	29 (47)	19 (53)	0.67
10	42 (43)	23 (37)	19 (53)	0.14
11	27 (28)	15 (24)	12 (33)	0.35
12	49 (50)	27 (44)	22 (61)	0.14
13	20 (20)	10 (16)	10 (28)	0.19
14	28 (27)	18 (29)	10 (28)	1.00
15	16 (16)	11 (18)	5 (14)	0.77
Mean (SD)	4.81 (2.16)	4.63 (2.28)	5.11 (1.92)	0.26^**^

*Statistical evaluation by the Chi-square test.

**Statistical evaluation by the independent T-test.

**Fig. 1:**
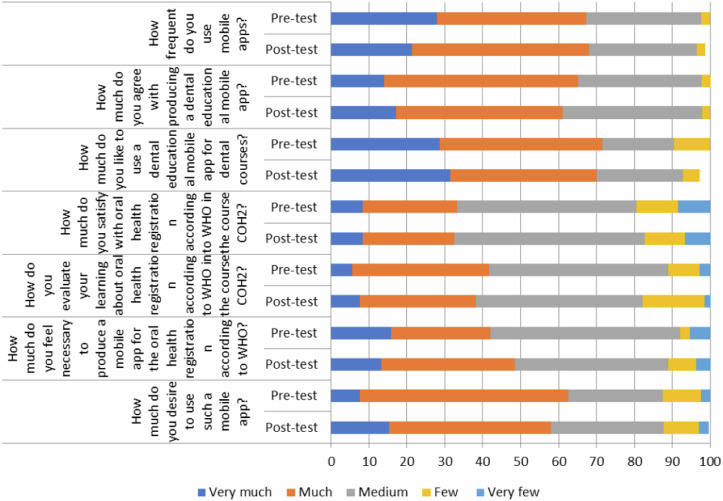
Distribution (%) of answers of the 7th semester students (intervention group) (*n* = 43) before and after the educational intervention to the questions related to the amount of use, skill and willingness to use applications based on mobile phones.

The comparison of responses from students in the intervention group concerning their mobile phone usage, proficiency in mobile technology, and interest in mobile application utilization, prior to and after the educational intervention, is illustrated in Fig. 1. It indicates no significant change in the percentage of students providing affirmative responses to the questions in the post-test compared to the pre-test.

Table 6 demonstrates the level of agreement among students in the intervention group concerning their attitude towards mobile phone-based learning, both before and after the educational intervention. It shows that, apart from the first statement, the differences in the percentage of students agreeing with the statements were not statistically significant.

**Table 6:** The percentage of agreement of the students of the intervention group (*n* = 43) (choosing the options completely agree and agree from the five-point Likert scale) with the statements regarding their attitude towards the use of mobile based learning separately in pre-test and post-test.

Item	Pre-test	Post-test	*p*-value*
I think m-learning is the most suitable environment for students with different learning styles (visual, auditory, learning by doing and experiencing, etc.).	56	77	0.01
I think that m-Learning can be more beneficial when it is combined with face to face learning in university courses. …	86	89	0.86
I think that M-Learning provides fast and practical learning.	86	80	0.53
I agree that M-Learning provides permanent learning.	35	66	0.41
I believe that it would be useful to spare my free time (Bus waiting, rest, etc.) with m-Learning outside of the course.	88	86	0.24
I find it interesting m-learning since I don’t want to carry books and course materials.	77	83	0.50
I think m-Learning is a good opportunity to improve my research skills.	79	79	0.93
Due to the potential dangers of the Internet (virus, etc.), I think m-Learning environment is unsafe.	14	14	0.95
I think mobile devices are not suitable for use in m-Learning environment since they need to be charged regularly.	5	14	0.47
I think m-Learning applications outside the classroom are useless because my attention is easily dispersed on the move.	19	20	0.38
I do not find it appropriate to use mobile devices in the classroom because they are harmful to human health.	5	11	0.11
I think M-Learning is not suitable for courses that require more reading and writing.	42	35	0.39
I like to participate in m-Learning as I can access course materials faster.	88	75	0.20
I believe that M-Learning supports planned and systematic study.	70	67	0.91
I believe that mobile tools are useful for taking notes in class.	54	69	0.09
I think mobile tools are useful for storing information.	95	94	0.74
I believe that mobile devices with large screens are useful for m-learning.	84	92	0.18
I think that M-Learning is an appropriate method for courses that require individual effort.	70	78	0.30
I prefer to learn m-Learning because I can compensate myself for the lessons I have missed.	81	83	0.92
I believe that M-Learning is useful for getting rapid feedback.	71	81	0.15
I believe that using m-Learning in the courses at the university will increase my freedom of learning.	74	78	0.78

*Statistical evaluation by the Mann-Whitney U test.

The analysis of the responses from students in the intervention group regarding their knowledge acquired from the book’s content, both prior to and following the educational intervention, is illustrated in Table 7. It is observed that the mean of correct responses (range from 1 to 15) in the post-test reflects a decline when compared to the pre-test; however, this alteration does not reach statistical significance. Ultimately, except for question #12, no statistically significant differences were found in the students’ responses between the pre-test and post-test.

**Table 7:** The frequency of correct answers and mean scores of students in the intervention group (*n* = 43) before and after the educational intervention to a set of fifteen multiple-choice questions from the book “Basic Methods of WHO Oral Health Examination; Fifth Edition, 2013”.

Question No.	Pre-test *n* (%)	Post-test *n* (%)	*p*-value*
1	3 (7)	1 (3)	0.62
2	24 (56)	22 (61)	0.65
3	8 (19)	2 (6)	0.10
4	8 (19)	10 (28)	0.42
5	11 (26)	11 (31)	0.80
6	14 (33)	12 (33)	1.00
7	32 (74)	19 (53)	0.06
8	26 (61)	19 (53)	0.50
9	5 (12)	10 (28)	0.08
10	12 (28)	12 (33)	0.63
11	28 (65)	19 (53)	0.35
12	26 (61)	10 (28)	0.006
13	6 (14)	5 (14)	1.00
14	33 (77)	22 (61)	0.14
15	10 (23)	10 (28)	0.79
Mean (SD)	5.72 (2.16)	5.11 (1.92)	0.19**

*Statistical evaluation by the Chi-square test.

**Statistical evaluation by Independent sample T-test.

## Discussion

Most of dental students in the present study spent a considerable time with their smartphone, were eager to use an educational application for the registration of oral health, showed positive attitude towards mobile learning and using mobile applications for educational purposes. This in accordance with the findings of a study among dental students in India [24]. On the other hand, the knowledge our dental students have gained out of the content of the book in both methods (self-read or mobile-based) were very limited. Furthermore, using a mobile application for teaching oral health registration to dental students in this interventional study showed no superiority to the traditional lecture-based education.

The participating students displayed a low level of proficiency in responding to knowledge questions, achieving an average score of 4.81 out of 15. In contrast, a considerable number of students expressed a positive attitude and enthusiasm for using mobile phones to learn dental courses. This contrast implies that the application developed in this study has not adequately met the educational needs of the students. This is in line with findings of a recent study which compared the efficacy of a smartphone-based application versus lecture-based learning for teaching of cephalometric landmark identification among dental students [25]. This may be due to failure to implement regular assessments and progress tracking within the mobile application which left the students without identifying their strengths and weaknesses. By providing personalized feedback and suggestions for improvement, students can actively work towards enhancing their understanding and knowledge in specific areas [26]. It is crucial to adapt and integrate new technologies to enhance the learning experience and improve knowledge retention. To address this issue, a comprehensive and innovative approach should be adopted, combining traditional teaching methods with mobile-based educational applications. By doing so, dental students can benefit from the convenience and accessibility of mobile learning while still receiving essential information from lectures and textbooks [27]. One potential solution is to develop an interactive mobile application that complements the curriculum and provides engaging content to aid in the learning process. This application could include interactive quizzes, visual demonstrations, and real-life case studies, allowing students to apply their knowledge in a practical setting [28].

Findings of the present study provide valuable insights into the distribution of students’ responses regarding their use of mobile apps in dental education. Analyzing these patterns and attitudes is essential for understanding the dynamics of incorporating mobile technology into dental curricula. High agreement percentages across both groups (93% in control and 92% in intervention) regarding the production of a dental educational mobile app indicate a positive inclination toward leveraging technology for educational purposes. This is in line with studies reflecting a broader trend observed in supporting the integration of technology in dental education [29–31].

While the current study suggests a higher mean knowledge score for the intervention compared to the control group, the difference was not statistically significant. This aligns with some studies indicating that innovative educational interventions, such as the use of mobile applications, may not always result in significantly higher academic performance [32].

The learning process for the students in both control and intervention in the present study could be matched to the Kirkpatrick model as follows:

For the control group:

1. Reaction level: Introduction of the book, collaborative translation, and creation of a powerpoint presentation can evoke a positive reaction from students. Engagement in discussions enhances the learning experience.
2. Learning level: Collaboration on translating sections of the book, creating a presentation, and engaging in discussions contribute to the acquisition of language and presentation skills. The dental examination provides hands-on learning in clinical forms and questionnaires.
3. Behavioral level: The hands-on dental examination where students fill out forms for their peers represents a behavioral change. Students apply theoretical knowledge to a practical, real-world scenario.
4. Impact level: The instructor collecting completed forms, evaluating them, and providing feedback represents the results level. It assesses the application of knowledge in a real context.

For the intervention group:

1. Reaction level: Introduction of a mobile application for reading and translating the book might elicit different reactions from students. Their response to using smartphones and technology for learning is part of the reaction level.
2. Learning level: Using the application for reading and obtaining translations contributes to language learning. Conducting dental examinations and completing questionnaires through electronic forms enhances technological and clinical skills.
3. Behavioral level: Entering data directly into electronic forms within the application represents a behavioral change. Students adapt to using digital tools for practical tasks.
4. Impact level: The results level is reached when the data entered the electronic forms is submitted to the instructor for evaluation and feedback. This stage assesses the application of knowledge in a digital context.

The current research presents several notable strengths. It effectively contrasts two distinct educational approaches: technology-enhanced learning via a mobile application and conventional self-directed learning activities. This comparative framework facilitates a deeper comprehension of how varying educational modalities influence student learning outcomes within the context of clinical education. The instructional materials were meticulously aligned with the undergraduate dental curriculum and derived from a reputable manual provided by the World Health Organization. This alignment guarantees that the educational content is pertinent, standardized, and directly relevant to the students’ prospective professional endeavors. Both participant groups undertook practical dental examinations, affording students essential hands-on experience. This experiential component not only enriches the learning process but also enables students to apply theoretical concepts in practical scenarios, thereby solidifying their grasp of oral health needs assessments. The incorporation of a mobile application aligns with the increasing shift towards digital learning in educational settings. The application not only grants access to educational resources but also facilitates immediate data entry and feedback, which can enhance student engagement and promote a more interactive learning environment. Furthermore, the utilization of questionnaires and clinical forms to evaluate knowledge and skills offers a comprehensive assessment of student performance.

There are several limitations associated with this study that warrant careful consideration: 1. The research findings are confined to a single dental school, which raises concerns regarding the broader applicability of the results. 2. The absence of randomization in the assignment of participants to intervention or control groups may lead to selection bias, as the intervention group was composed of students in their 7th semester, while the control group included those in their 8th semester. This disparity in academic standing could lead to differences in prior knowledge, motivation, and overall skill levels, potentially affecting the study’s outcomes. 3. The lack of blinding in the study design raises the possibility of performance and detection bias. Performance bias refers to the risk associated with the awareness about the intervention being applied. Since students in the intervention or control group were aware about the way of teaching, this may lead them to an unintentional or deliberate perception and report of a more favorable outcome. On the other hand, detection bias pertains to the risk of bias in the evaluation of outcomes. In the present study awareness of the supervisor about the method of teaching in both groups may inadvertently or intentionally influence his evaluations.

There is a need for further investigation into the long-term effectiveness of smartphone applications in enhancing dental students’ knowledge and skills in oral health registration. Future research could also examine the optimal design and features of these applications, as well as their comparative effectiveness with other technology-based teaching methods such as virtual reality or augmented reality. Given the rapid advancements in technology, future studies could explore the integration of artificial intelligence or machine learning algorithms in these applications for teaching oral health registration.

## Conclusion

Findings of the current study failed to show higher effectiveness for a smartphone application comparing to the traditional teacher-centered teaching method. This raises questions about the role of mobile applications in education, highlighting the need for further research on how technology can improve learning in relation to teaching strategies. The students’ considerable time devoting to mobile usage and their positive attitude towards mobile-based learning, however, are promising for further research to explore the full potential of these applications in dental education.

While the overall students’ output from the present study suggests a positive trend, the variability in individual question outcomes emphasizes the need for a distinctive understanding of the impact of such interventions. Further research, perhaps incorporating qualitative assessments and exploring the specific elements of the intervention contributing to learning outcomes, can provide a more comprehensive understanding of technology-enhanced education in dental settings.

## Data Availability

The datasets generated and/or analyzed during the current study are available from the corresponding author on reasonable request.
